# Appraisal of postbiotics in cancer therapy

**DOI:** 10.3389/fphar.2024.1436021

**Published:** 2024-09-20

**Authors:** Aruna Senthil Kumar Sudaarsan, Asit Ranjan Ghosh

**Affiliations:** Department of Integrative Biology, School of Bio Sciences and Technology (SBST), Vellore Institute of Technology (VIT), Vellore, Tamil Nadu, India

**Keywords:** cancer, probiotics, postbiotics, gut microbiota, cell-free supernatants, bacteriocins, conjugated linoleic acid, exopolysaccharides ∗ NA-not approved

## Abstract

Cancer remains a multifactorial disease with an increased mortality rate around the world for the past several decades. Despite advancements in treatment strategies, lower survival rates, drug-associated side effects, and drug resistance create a need for novel anticancer agents. Ample evidence shows that imbalances in the gut microbiota are associated with the formation of cancer and its progression. Altering the gut microbiota via probiotics and their metabolites has gained attention among the research community as an alternative therapy to treat cancer. Probiotics exhibit health benefits as well as modulate the immunological and cellular responses in the host. Apart from probiotics, their secreted products like bacteriocins, exopolysaccharides, short-chain fatty acids, conjugated linoleic acid, peptidoglycan, and other metabolites are found to possess anticancer activity. The beneficiary role of these postbiotic compounds is widely studied for characterizing their mechanism and mode of action that reduces cancer growth. The present review mainly focuses on the postbiotic components that are employed against cancer with their reported mechanism of action. It also describes recent research works carried out so far with specific strain and anticancer activity of derived compounds both *in vitro* and *in vivo*, validating that the probiotic approach would pave an alternative way to reduce the burden of cancer.

## Graphical Abstract

**Figure:**
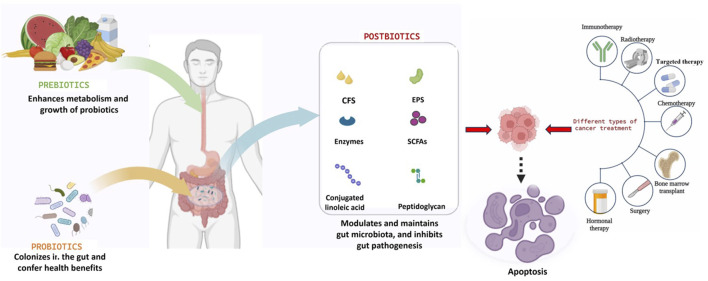

## 1 Introduction

Cancer remains one of the main causes of mortality and creates despair in the human community across the globe (Sung et al., 2021). Along with the growth of population and aging, cancer continues to exist as the leading determinant of mortality compared to heart diseases (Bray et al., 2018). Cancer denotes the uncontrolled growth of cells following the spread to distant organs by metastasis (Ghosh and George, 2023). It is mainly characterized by altered cell signaling and metabolism leading to countless proliferation (Upadhyay, 2020). International Classification of Diseases for Oncology has classified cancer into six main groups based on tissue types, namely, carcinoma, lymphoma, sarcoma, myeloma, leukemia, and mixed type. Even though there are more than 100 types of cancer, the most common cancer type includes breast cancer with an incidence rate of 11.7%, followed by lung cancer at 11.4%, colon cancer at 10%, prostate cancer at 10%, and finally the stomach cancer with 5.5% (Sathishkumar et al., 2022). Compared to other diseases, there are very few treatments for cancer including surgery, chemotherapy, immunotherapy, and radiation therapy (Debela et al., 2021). Despite advances in diagnostics and therapeutics, the number of cancer cases has been increasing in the past 2 decades (Falzone et al., 2018). Reduction in cancer mortality mainly relies on continuous progress in pharmacological fields and the introduction of effective drugs and therapies (Miller et al., 2016).

Human gut harbors trillions of microbes like bacteria, fungi, and yeast that execute favorable tasks to the host (Thursby and Juge, 2017). The microbiota favors the host through a wide range of functions like strengthening and shaping the intestinal epithelium, acting against harmful pathogens, regulating host immunity, and also a part in energy scavenging (Natividad and Verdu, 2013; Gensollen et al., 2016). Gut microbiota has been considered an important organ, due to its communicational axis with the rest of the host’s organs through humoral, endocrine, neural, and metabolic pathways (Ahlawat and Sharma, 2021). Gut microbiota depends on several factors like human lifestyle, age, environmental factors, and diet as it can modulate the microbiome (Afzaal et al., 2022). However, dysbiosis occurs when intestinal bacterial growth and related metabolism are disintegrated. Dysbiosis is the disproportion of the microbial composition that results in the alteration of bacterial metabolic activities in the human gut (DeGruttola et al., 2016). Dysbiosis can result in developing inflammation in the gastrointestinal tract (GIT), diarrhea, neurodegenerative disorders, and even cancer (Asseri et al., 2023). Numerous studies have concluded that dysbiotic microbiome and its derivatives are associated with the cause of inflammatory diseases like IBDs (inflammatory bowel diseases), CVDs (cardiovascular diseases), CKDs (chronic kidney diseases), NAFLD (non-alcoholic fatty liver diseases), and cancer (Afzaal et al., 2022). Therefore, the role of homeostatic gut microbiota and their metabolites play a significant role in human health which has directed researchers to investigate the connections of microbes in metabolism (Cardona and Roman, 2022). The prokaryotic members in a healthy gut are generally friendly and beneficial to health and are probiotics.

Probiotics are beneficial microorganisms that utilize dietary fibres, prebiotics, and secrete metabolites known as postbiotics (Kim S. et al., 2021. Generally, probiotics can be consumed by integrating them into foods like dairy products or non-dairy food forms as additional supplements (Latif et al., 2023). Fermented foods that are consumed comprise active microbes closely similar to the strains regarded as probiotics. These fermented foods enhance their nutritional value by converting substrates into bioactive metabolites (Marco et al., 2017). There are expanding shreds of evidence favoring the beneficial effects of probiotic consumption, including maintenance of gut health, improved immune response, and cancer prevention (Kechagia et al., 2013). Probiotics are widely known as a potential therapeutic agent against many diseases like necrotizing enterocolitis (NEC), acute infectious diarrhea, antibiotic-associated diarrhea (AAD), upper respiratory infections, irritable bowel syndrome (IBS), gastroenteritis, vaginal candidiasis, ulcerative colitis, traveler’s diarrhea, and various allergic diseases (Hawrelak, 2003; Wang et al., 2019; Kumar et al., 2024). Apart from probiotics, postbiotics have been reported to possess health benefits to hosts with several properties including infection control and prevention, induction to apoptosis, mitigation of inflammation, immunomodulation, and reinstating of eubiosis. Postbiotics refer to the bioactive molecules that are released from probiotics as a result of fermentation and cell lysis (Kim Y. J. et al., 2021). Recent research findings have validated the effect of postbiotic components against several life-threatening diseases and disorders.

Current management of cancer treatment involves standard drugs that not only act against cancer cells but also affect normal cells developing resistance towards them and likely related therapies remain under challenge (Raguz and Yagüe, 2008). Side effects are the common outcome of cancer treatment (chemo- and radiotherapy) which reduces the quality of patients’ lives and increases agonies. Probiotics and prebiotics-mediated therapeutics showed promising results in mitigating such unwanted side effects. A study analyzed 20 published clinical trials with probiotics where 17 trials experienced beneficial impact in reducing side effects and three did not show visible results (Rodriguez-Arrastia et al., 2021). Due to chemo- and radio-therapy, non-cancerous normal cells are also induced to malfunction physiologically which may lead to bleeding, anemia, loss of taste, nausea, diarrhea, inflamed mucus, dysbiosis, and many more discomforts (Akbarali et al., 2022). To overcome such situations, there is continuous research to use probiotics as an adjuvant if not directly for cancer therapy. Research outcome shows acceptable information in several clinical trials [Renzis et al., 2007,]. The use of probiotics in mitigating side-effects developed in patients due to cancer treatment is enlisted in Table 1, demonstrating the type of treatment offered to patients with different cancer types and the relative improvement of patient’s quality of life. On the other hand, there is a long list of probiotics that are mostly used as a dietary supplement to maintain good health from different conditions (gas, constipation, diarrhea, oral thrush, IBS, urinary tract infection, vaginal pH imbalance, etc*.*) though none of the listed drugs is FDA approved but approved by similar other organizations across countries, like World Organisation of Gastroenterology, Therapeutic Goods Administration (TGA), Ministry of Health Malaysia, and Chinese regulatory authority, the State Food and Drug Administration (SFDA) and commercially viable (Table 1) [https://www.drugs.com/drug-class/probiotics.html].

**TABLE 1:** List of approved and non-approved commercial probiotics employed as adjuvants to cancer therapies.

S.NO	Approved by	Commercial name	Probiotic strains	Cancer type	Cancer therapy	No. of patients	Time of dosage	CFU	Inference	References
1	World Organisation of Gastroenterology	VSL-3	*L. casei, L. plantarum, L. acidophilus, L. delbruekii subsp. bulgaricus, B. longum, B. breve, B. infantis, Streptococcus salivarius subsp. thermophilus*	Sigmoid, rectal, and cervical	Radiotherapy	243/490	From the beginning to the end of radiation therapy	112.5 billion	Reduction in radiation-induced diarrhea, no case of bacteremia, and lessened intestinal toxicity	Renzis et al. (2007)
2	Therapeutic Goods Administration (TGA), Australia	Infloran	*B. bifidium* NCDO 2203, *L. acidophilus* NCDO1748	Cervical cancer	Radiotherapy and chemotherapy	32/63	One week before radiotherapy and till the end	2 billion	Reduction in radiation-induced diarrhea, anti-diarrheal, and improved stool consistency	Chitapanarux et al. (2010)
3	Ministry of Health Malaysia (MOH)	Hexbio MCP	*L. acidophilus* BCMC 12130, *L. casei* BCMC 12313, *L. lactis* BCMC 12451, *B. bifidum* BCMC 02290, *B. longum* BCMC 02120 and *B. infantis* BCMC 02129	Colorectal cancer	Chemotherapy	70/160	Patients underwent chemotherapy	30 billion	Reduced the side effects due to chemotherapy and restoration of the integrity of intestinal cells	Golkhalkhali et al. (2018)
4	Ministry of Health Malaysia (MOH)	Hexbio MCP	*L. acidophilus* BCMC 12130, *L. casei* BCMC 12313, *L. lactis* BCMC 12451, *B. bifidum* BCMC 02290, *B. longum* BCMC 02120 and *B. infantis* BCMC 02129	Colorectal cancer	Post-surgery	30/75	Patients after the surgery	30 billion	Reduction the level of pro-inflammatory cytokines TNF-α, IL-17A, IL-17C, IL-22, IL-10 and IL-12 and other complications after the surgery	Zaharuddin et al. (2019)
5	Chinese regulatory authority, the State Food and Drug Administration (SFDA)	Bifico	*Bifidobacterium longum, Lactobacillus lactis*, and *Enterococcus faecium*	Nasopharyngeal cancer	Chemoradiotherapy	64/99	Patients underwent chemotherapy	3 billion	Reduction in oral mucositis	Jiang et al. (2019)
6	*NA	Bifilact	*L. acidophilus* LAC-361 and *B. longum* BB-536	Pelvic cancer	Post-surgery, Radiotherapy and chemotherapy	86/140	Patients who underwent radiotherapy	1.3 billion	Reduction in severe diarrhea and average bowel movement	Demers et al. (2014)
7	NA	Biscanen (Capsules)	*Bacillus licheniformis*	Gynecological and urological cancers	Radiotherapy	124/248	Two weeks before the start	250 million	Prevention of radiation-induced enteropathy	Kim S. et al. (2021)
8	NA	*L. casei* DN-114 001	*L. casei* DN 114001	Gynecological cancer	Radiotherapy and chemotherapy	30/45	Undergoing therapy	10^8^ CFU	Effect on stool consistency rather than reduction in radiation-induced diarrhea	Giralt et al. (2008)
9	NA	Biogurt	*L. acidophilus* LA-5 plus *B. animalis subsp. lactis* BB-12	Cervical cancer	Radiotherapy and chemotherapy	26/74	During radiotherapy treatment	1.75 billion	Reduction in the incidence of radiation-induced diarrhea, and reduced the usage of loperamide for the prevention of diarrhea	Linn et al. (2019)
10	NA	Golden Bifid	*Bifidobacterium* (ATCC 15697), *L. bulgaricus* (ATCC 11842) and *S. thermophilus* (ATCC 19987)	Pelvic cancer	Radiotherapy	24/46	During radiotherapy treatment	60 million	Reduction in abdominal pain and diarrhea	Shao et al. (2014)
11	NA	SiLiankang	*B. infantis, L. acidophilus, E. faecalis* and *B. cereus*	Cancer	Chemotherapy	48/100	Patients underwent chemotherapy	50 billion	Normal bowel movement and lessened constipation caused by chemotherapy	Liu and Huang (2014)
12	NA	Antibiophilus	*L. rhamnosus*	Abdominal cancer	Radiotherapy	102/205	Patients underwent chemotherapy	1.5 billion	Reduction in diarrhea, normal fecal consistency and bowel movements	Urbancsek et al. (2001)
13	NA	Gefilus	*L. rhamnosus* GG (ATCC 53103)	Colorectal cancer	Chemotherapy	98/150	Patients underwent chemotherapy	5 billion	Reduction in frequency of diarrhea	Österlund et al. (2007)
14	NA	*L. brevis* CD2 lozenges	*L. brevis* CD2	Head and neck cancer	Radiotherapy and chemotherapy	100/200	Patients who underwent chemo and radiotherapy	2 billion	Reduction in the incidence of oral mucositis caused by chemo-radiotherapy	Sharma et al. (2012)
15	NA	*L. brevis* CD2 lozenges	*L. brevis* CD2	Leukaemia	Chemotherapy	30	Patients underwent chemotherapy	2 billion	Reduction in the oral mucositis condition	Sharma et al. (2016)

^*^ NA- Not Approved

In the act of preventing and treating cancer, probiotics are employed due to their pivotal role in host interactions and conferring health benefits (Legesse Bedada et al., 2020). Since the last centennial, probiotics and their derived metabolites (components of postbiotics) have set up the cornerstone of research against all types of cancer (Nazir et al., 2018). Most reviews have mainly focused on the role of probiotics against colorectal cancer (CRC) however the current discussion reviews exclusively on postbiotics with a general view of probiotics, prebiotics, postbiotics, next-gen probiotics and their preventive roles on different cancers with plausible explanations of underlying mechanisms of action and future directions.

## 2 Probiotics, prebiotics, postbiotics, and synbiotics

**FIGURE 1:**
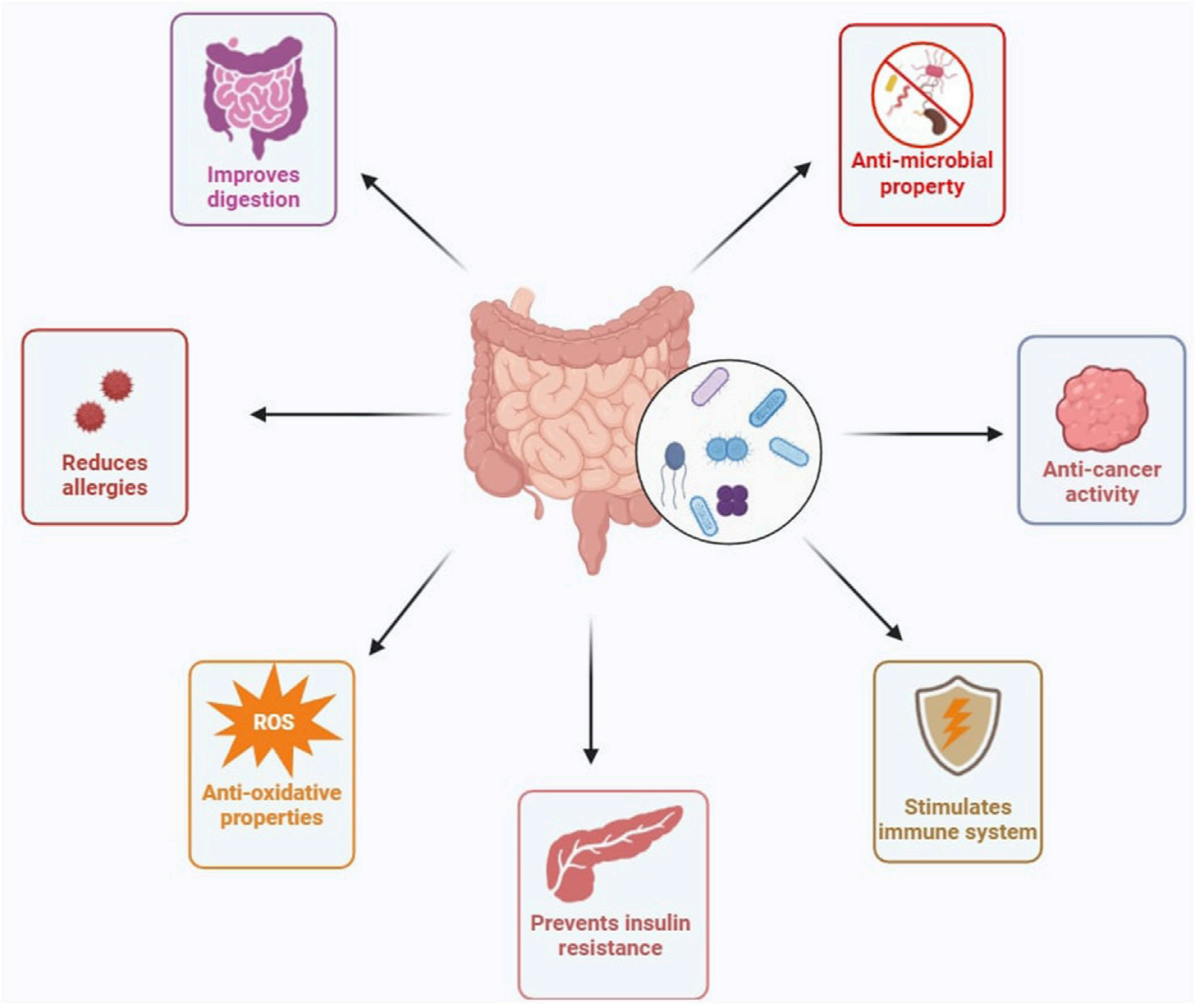
Various effects of probiotics on host’s health. (Figures were generated using BioRender.com)

According to the Food and Agriculture Organization of the United Nations (FAO) and the World Health Organization (WHO) in 2001, “Probiotics are live microorganisms which, when administered in adequate amounts, confer a health benefit on the host” (Hill et al., 2014; Indian Council of Medical Research Task ForceCo-ordinating Unit ICMRCo-ordinating Unit DBT, 2011). Bacterial strains in the genera of *Lactobacillus, Lactococcus, Bacillus, Enterococcus, Pediococcus, Streptococcus,* and *Propionibacterium* are considered to be potential probiotic microbes (Hamad et al., 2022). Among them, lactic acid-producing bacteria [LAB] and Bifidobacteria have been explored for a wide range of applications (Figure 1) (Bron et al., 2011). Probiotics produce various products like antimicrobial substances, exopolysaccharides (EPS), short-chain fatty acids (SCFAs), conjugated linoleic acids (CLA), and other metabolites during metabolism, which are directly involved in the benefit of human health (Marco et al., 2017). Probiotics affect the immune responses that are intervened by various immune cells like B and T lymphocytes, dendritic cells, macrophages, and natural killer (NK) cells (Kerry et al., 2018). The innate immune system of the host has been studied against its link to probiotics and revealed that expression of cytokines presented by antigen-presenting cells, augmenting type 1 helper T cell response, and finally activation of natural killer cells (Ashraf and Shah, 2014). Additionally, these probiotic bacteria can have the ability to influence the nervous system by communicating via the gut-brain axis (Morkl et al., 2020). Probiotics are thus considered functional foods with scientific proofs which validate beneficial properties by producing bioactive metabolites for modulating gut microbiota, and immunomodulation (Lin, 2003). Apart from health benefits, the widespread usage of live probiotics is associated with some unwanted health effects among children and adults (Doron and Snydman, 2015). Another concern about using live probiotics is that they might get transported into blood vessels and neighbouring tissues resulting in bacteremia in immunocompromised individuals (Kataria et al., 2009). Similarly, other issues with live probiotics may include the transfer of antibiotic-resistant genes in the human gastrointestinal tract (GIT) (Mater et al., 2008). However, the health-beneficial realms of probiotics and their derivatives are so big and effective, that these demerits are negligible.

For the past 2 decades, postbiotics have acquired more attention from researchers to explore their potential applications in medicine (Ali et al., 2023). Postbiotics are defined as the preparation of inanimate probiotics (para probiotics, ghost probiotics), their fermented metabolites, and structural components that confer health benefits on the host (Salminen et al., 2021). The International Scientific Association of Probiotics and Prebiotics (ISAPP) defined postbiotics are “preparations of inanimate microorganisms and/or their components that confer health benefit on the host.” Postbiotic preparations contain probiotic-derived components such as cell lysates, metabolites, peptides, enzymes, vitamins, proteins, exopolysaccharides, and extracellular vesicles (Deshpande et al., 2018). Studies on health beneficial properties of postbiotics reveal that postbiotics possess characteristics and are more advantageous than live probiotics. Probiotics need support for assured shelf life while postbiotics need not. However, probiotics can colonize, and antagonize pathogens by interacting with the host system. On the other hand, postbiotics can pass through the mucous layer quickly, with no risk of infection in immunocompromised individuals, no possibility of antibiotic resistance gene transfer, and are convenient to standardize transport and storage (De Marco et al., 2018). Postbiotics therefore own better choices of their applications in developing several functional foods than probiotics (Vinderola et al., 2022).

On the other hand, prebiotics is another term used for food ingredients like non-digestible, resistant starch, and fibers in human GIT and are good for the growth of gut microbiota. Prebiotics are defined as a “selectively fermented ingredient that results in changes in the composition and activity of gastrointestinal microbiota, conferring benefits upon host health” (Gibson et al., 2017). These ingredients are not digested by humans but become the source of energy harvest, growth, and metabolite production by gut microbiota. Thus, it can modify the gut microbiome and influence the host’s health condition (Rastall and Gibson, 2015). Most of the prebiotics belong to the class of carbohydrates that are present naturally in the human diet (Slavin, 2013). Common prebiotics include oligofructose, inulin, fructo-oligosaccharides (FOS), galactose-oligosaccharides (GOS), and xylose-oligosaccharides (XOS) (Hutkins et al., 2016). These are obtained from natural resources like fruits, vegetables, and grains that are commonly used in day-to-day life. Prebiotics have been reported to reduce the prevalence of diarrhea, irritable bowel syndrome, and even colon cancer (Peña, 2007). Despite this, prebiotics were identified to enhance the bioavailability and uptake of nutrition, and suppression of risk factors of cardiovascular diseases (Pokusaeva et al., 2011). These components are stable, temperature resistant, and can thrive in stomach acids, but may lead to gastrointestinal discomfort (Marteau and Seksik, 2004). Considering differences among probiotics, prebiotics, and postbiotics, all three have functional relationships for the promotion of health benefits of the host (Ji et al., 2023).

Synbiotics refers to the complex mixture of both prebiotics and probiotics formulation to improve human health (Markowiak and Śliżewska, 2017). According to ISAPP, synbiotics are of two types: complementary and synergistic. Synergistic synbiotics consist of a substrate that is specifically utilized by a co-administered microbe, whereas in complementary synbiotics both probiotics as well as prebiotics together confer health benefits independently (Swanson et al., 2020). Several studies have reported that synbiotics stimulate health and nutrition in the host (Yadav et al., 2022). Synbiotics were found to reduce the risk of CVDs and insulin resistance in aged individuals (Cicero et al., 2021).

## 3 Postbiotics-an endowing anticancer agent

**FIGURE 2:**
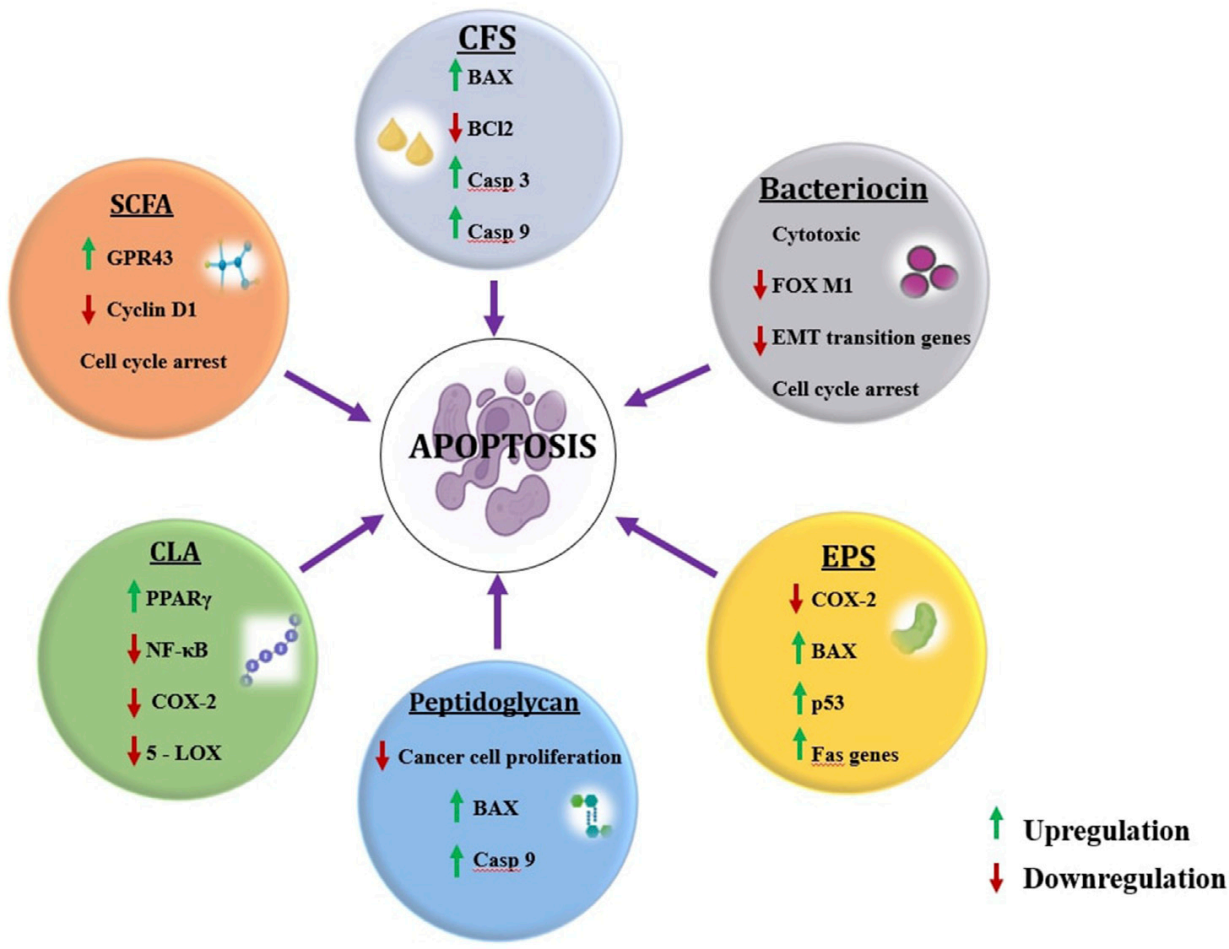
Postbiotic components and their roles in mitigating cancer. (Figures were generated using BioRender.com)

The concept of postbiotics mounted during this decade however terms like postbiotics, paraprobiotics, and fermented infant formulas (FIFs) came into existence in 1986 with increasing growth of research and development, as reviewed elsewhere (Wegh et al., 2019). The postbiotic is a pool of functional components that include cell-free supernatants (CFS), short-chain fatty acids (SCFA), peptides, bacteriocins, exopolysaccharides (EPS), biosurfactants, conjugated linoleic acid (CLA) and peptidoglycans (PG) (Figure 2).

### 3.1 Cell-free supernatants

Cell-free supernatant (CFS) of probiotic strains has driven interest among researchers in finding bioactive molecules targeting various health problems. Generally, CFS is a fluid that consists of nutrients utilized in the growth medium and metabolites released as a result of microbial development (Lee et al., 2022). CFS of probiotics contains low molecular weight compounds like hydrogen peroxide, and organic acids and high molecular weight compounds like bacteriocins (Kapustian et al., 2018). These components are obtained from centrifugation of the grown cultures directly followed by filtration. The composition of CFS may vary with the supplementation in the base culture medium like MRSB (de Man Rogosa, and Sharpe broth). CFS has been explored widely for its anti-microbial, anti-biofilm, anti-inflammatory, and anti-cancer studies. The results obtained through numerous studies report that CFS of probiotic bacteria is one of the promising biotherapeutic agents to treat a wide range of diseases including cancer (Żółkiewicz et al., 2020).

As CFS of probiotic bacteria contains a wide range of metabolites, they are employed in studying many types of cancer. There are several studies carried out with the involvement of CFS of *Lactobacillus* spp.*, Bifidobacterium* spp.*, Bacillus* spp.*, Enterococcus* spp.*, Pediococcus* spp.*, Lactiplantibacillus* spp.*,* and *Saccharomyces* spp. using different cancer cell lines and induced animal models against cancers such as colon, breast, gastric, cervical, skin, and lung cancer (Table 2). Results obtained from these *in vitro* and *in vivo* studies suggest that CFS bears therapeutic potential in reducing tumor growth and inducing apoptosis. However, the proper mechanism of action of CFS against cancers and further phase trials need to be elucidated.

**TABLE 2:** Effects of probiotic CFS against cancer.

Year	Probiotic strain	Cancer type	Study subject (cell lines/test animals)	Outcome	References
2008	*Bifidobacterium adolescentis* SPM0212	Colon cancer	HT29, SW480 and Caco-2	Inhibition of enzymes like tryptophanase, potentiate TNF-α production	Kim et al. (2008)
2010	*Bacillus polyfermenticus*	Breast cancer	HT-29, Caco-2, and DLD-1	Downregulation of transcription factor E2F-1, Suppression of ErbB2 and ErbB3 genes	(Ma et al., 2010)
2012	*Lactobacillus rhamnosus* GG	Colorectal cancer	HCT-116	Decreases metastasis in colon cancer cells	Escamilla et al. (2012)
2014	*Lactobacillus delbrueckii*	Colon cancer	SW620	Downregulation of MMP-9, Inhibition of proliferation through apoptosis	Wan et al. (2014)
2015	*Bacillus polyfermenticus* KU3	Multi cancer approach	HeLa, LoVo, HT-29, AGS, MCF-7	Decrease in production of proinflammatory cytokines and nitric oxide	(Lee et al., 2015)
	*Lactobacillus fermentum* NCIMB5221	Colorectal Cancer	SW-480, Caco-2 and CRL-1831	Induction of apoptosis in CRC cells	Meenakshi (2015)
	*Enterococcus lactis* IW5	Multi cancer approach	HeLa, MCF-7, AGS, HT-29, Caco-2	Inhibition of tumor growth and downregulation of ErbB2 and ErbB3 genes	Nami et al. (2015)
2016	*Lactobacillus casei* ATCC 393	Colon Cancer	Murine CT26 and human HT29	Upregulation of TRAIL gene and downregulation of Survivin	Tiptiri-Kourpeti et al. (2016)
	*Lactobacillus reuteri* NCIMB 701359	Colon cancer	SW480, Caco-2	Induction of apoptosis and inhibition of cancer cell proliferation	Kahouli and Handiri (2016)
	*Lactobacillus pentosus* B281, *L. plantarum* B282	Colorectal cancer	Caco-2 and HT-29	Downregulation of cyclin genes and cell cycle arrest in G1-phase	Saxami et al. (2016)
2017	*Bacillus coagulans*	Colon cancer	COLO 205	Upregulation of BAX gene, downregulation of Bcl2 gene, increased production of Cytochrome c, and induction of apoptosis	Madempudi and Kalle (2017)
2018	*Lactobacillus casei* (SR1,SR2)*, Lactobacillus paracasei* (SR4)	Cervix cancer	HeLa	Upregulation of BAX, BAD, Casp3, Casp9 Downregulation of the Bcl-2 gene	Riaz Rajoka et al. (2018)
2019	*Lactobacillus acidophilus, Lactobacillus delbrueckii*	Colon cancer	HT-29	Upregulation of Caspase-3 and Caspase-9 gene expression, upregulation of Bax/Bcl-2 gene	Baghbani-Arani et al. (2020)
2020	*Lactobacillus acidophilus* CICC 6074	Colon cancer	HT-29, 32 BALB mice	Upregulation of the Bax gene, the release of Cyt c by activating mitochondrial pathway	Guo et al. (2020)
	*Lactobacillus salivarius*	Colorectal cancer	HT-29	Downregulation of cyclin D1, cyclooxygenase-2, and protein kinase B	Dong et al. (2020)
	*Lactobacillus fermentum*	Colon cancer	DLD-1, HT-29, WiDr	Induces apoptosis, inhibits NF- κB activation	(Lee et al., 2020)
	*Lactobacillus plantarum* L-14	Skin cancer	A375	Downregulation of the Bcl-2 gene, cleavage of caspase-9, caspase-3, and PARP, induction of apoptosis through the intrinsic pathway	Park et al. (2020)
	*Lactobacillus reuteri*	Colon cancer	HT29-ShE	Downregulation of MMP-9 and COX-2. Upregulation of	Maghsood et al. (2020)
	*Lactobacillus plantarum* YYC-3	Colon cancer	Caco-2 and HT-29	Downregulation of MMP2, MMP9, and suppression of the VEGF pathway	Yue et al. (2020)
2021	*Pediococcus acidilactici*	Colon cancer	Caco-2 and HT-29	Downregulation of TNF- α, and upregulation of IL-10	Barigela and Bhukya (2021)
	*Lactobacillus fermentum* ZS09	Colon cancer	4-week-old C57BL/6 mouse - 60	Inhibition of EMT by regulating Wnt/β-catenin pathway	Liu et al. (2021)
	*Odoribacter splanchnicus*	Colorectal cancer	HCT 116, CRC mouse model	Inhibition of CRC cell proliferation, amelioration of tumorigenesis in allograft mice model of CRC	Oh et al. (2021)
	*Lactobacillus acidophilus ATCC4356*	Breast cancer	MCF-7, MCF-7 Xenograft mouse model	Inhibition of cell proliferation and reduction in weight of tumor	Behzadi et al., 2021
	*Lactiplantibacillus plantarum* L125	Colon cancer	HT-29	Anti-clonogenic and anti-migration effect	Tegopoulos et al. (2021)
2022	*Lactobacillus plantarum* IIA-1 A5, *Lactobacillus acidophilus* IIA-2B4	Colon cancer	WiDr	Dose-dependent anti-cancer activity	Adiyoga et al. (2022)
	*Faecalibacterium prausnitzii*	Colorectal cancer	HCT116	Inhibition of activation of NF-KB, increased production of IL-10	Dikeocha et al. (2022)
	*Lactiplantibacillus plantarum*	Colon cancer, Cervical cancer	Caco-2, HeLa	Induction of apoptosis in Caco-2 cell line, induction of hydrogen peroxide production and induction of ROS	Nowak et al. (2022)
	*Lactobacillus rhamnosus* SD1, SD4, SD11, GG	CRC	Caco-2, HIEC-6	Increased expression of IL-10 and hBD(2–4)	Pahumunto and Teanpaisan (2023)
	*Saccharomyces boulardii*	Breast cancer	MCF-7, MCF-7/MX	Suppression of Survivin gene expression, induced apoptosis	Pakbin et al. (2023)
2023	*Lactiplantibacillus plantarum* OC01	CRC	HCT 116, HT 29	Suppression of IL-6 limits cancer growth and progression	Vallino et al. (2023)
	*Escherichia coli* 536	Bladder cancer	Bladder cancer cell line 5637	CX3CL1 gene which plays a role in the elimination of neoplastic cells decreased and CCL2, a gene that promotes lymphatic metastasis was found to be downregulated	Mehmandar-Oskuie et al. (2023)
	*Lactobacillus bucheri*	Gastric cancer	AGS	Increased expression of BAX, CASP9, and CASP3	Abedi et al. (2023)
	*Lactobacillus rhamnosus* GG	Colon cancer, Metastatic melanoma	HCT-116, Caco-2, HT-29 and A375	Mitotic arrest in the G2/M phase of cell cycle leads to reduction in viability	(Salemi et al., 2023)
	*Enterococcus faecalis* KUMS-T48	Gastric cancer, Colon cancer	HT-29	Downregulation of IL-1β, Upregulation of IL-10 gene expression in HT-29 cell line	Salek et al. (2023)
	*Saccharomyces boulardii*	Gastric cancer	AGS	Downregulation of Survivin, NFκB, IL-8 genes	Pakbin et al. (2023)
	*Lactobacillus plantarum* ATCC 14917, *Lactobacillus rhamnosus* ATCC 7469	Colon cancer	Caco-2, HUVEC	Downregulation of anti-apoptotic genes Bcl-2 and Bcl-xl	Amin et al. (2023)
2024	*Lactobacillus casei* CRL431	CRC	HCT-116 and HT-29	Arrested cell cycle (G0/G1) phase	Abbasi et al. (2023)
	*Pediococcus acidilacti*	Breast cancer	MDA-MB-231	Upregulation of TWIST1 gene involved in EMT, reduction in cancer cell motility	Adumuah et al. (2024)
	*Lactiplantibacillus plantarum*	Melanoma, Breast cancer	HaCaT, A375, MCF-7	Upregulation of BAX, downregulation of Bcl-2	Budu et al. (2024)

### 3.2 Bacteriocins

Bacteriocins are cationic peptide molecules that are produced by all classes of bacteria. These bioactive components are found to be heat-stable, proteases-sensitive, synthesized ribosomally, and have different modes of action (Darbandi et al., 2022). Bacteriocins have been classified into three classes I, II, and III by their structural and physio-chemical properties (Zacharof and Lovitt, 2012). Class I bacteriocins are known as lantibiotics and are small molecular weight peptides ranging from <5 KDa. Apart from lower molecular weight class I are heat stable and contains amino acids like lanthionine, and methyllanthionine. Further classification of class I bacteriocins is based on the charge associated and the mode of action. Class I lantibiotics are classified into two types, Type A lantibiotics (Ex: nisin, positively charged, causes pore formation in cell membrane on the target species) and type B lantibiotics (Ex: Mersacidin, negative charge, interferes in cell wall synthesis of target species) (Kaur and Kaur, 2015). Class II bacteriocins are <10 kDa, heat stable, and are commonly known as non-lanthionine peptides. Further classification of class II bacteriocins is subclass IIa (monomeric), IIb (Contains two segments), and IIc (circular bacteriocins) (Cotter et al., 2005). Class III are the high molecular weight heat liable proteins ranging around >30 kDa like colicins and enterolysin (Kaur and Kaur, 2015).

Several studies have carried out trials on bacteriocins against cancer. Factors that selectively act against cancer cells have been observed in numerous studies however, no proper mechanism of action could be proposed. Cancer cells are characterized by a lack of asymmetry in phospholipid types, and possess a negative charge due to the presence of anionic phosphatidylserine, glycosylated mucins, heparin, and gangliosides (Riedl et al., 2011). Bacteriocins are cationic peptides that can bind to the negatively charged membrane of cancer cells and act against them (Hoskin and Ramamoorthy, 2008). Here we have listed the studies where bacteriocins derived from probiotic bacterial strains were employed against types of cancer (Table 3).

**TABLE 3:** Role of probiotic-derived bacteriocin against cancer.

Year	Probiotic strain	Bacteriocin	Cancer type	Study subject (cell lines/test animals)	Outcome	References
2008	*Streptomyces azureus* ATCC 149215	Thiostrepton	Breast cancer	MCF-7	Downregulation of the FOXM1 gene responsible for the proliferation and development of tumor	Kwok et al. (2008)
2011	*Pediococcus acidilactici* K2a2-3	Pediocin K2a2-3	Colon cancer, cervical cancer	HT29, HeLa	Cytotoxic to both HT29 and HeLa cell lines	Villarante et al. (2011)
2012	*Lactococcus lactis*	Nisin	HNSCC	UM-SCC-17B, UM-SCC-14A, HSC-3	Activation of CHAC1(apoptotic mediator) and decrease cell proliferation	Joo et al. (2012)
	*Pediococcus acidilacti* MTCC5101	Pediocin CP2	Multicancer approach	HepG2, HeLa, MCF7, Sp2/0-Ag14	Cytotoxic activity against cancerous cell line	Kumar (2012)
2013	*Lactobacillus fermentum* HV6b MTCC10770	Fermenticin HV6b	Multicancer approach	HepG2, HeLa, MCF7, Sp2/0, HEK-293	Cytotoxic activity and induction of apoptosis in cancer cell lines	Kaur et al. (2013)
2015	*Lactococcus lactis*	Nisin ZP	HNSCC	UM-SCC-17B, UM-SCC-14A, HSC-3, OSCC-3	Induction of apoptosis by calpain activation in a dose-dependent manner, apoptosis in endothelial cells, and decreased cell proliferation	Kamarajan et al. (2015)
	*Lactococcus lactis*	Nisin	Skin cancer	Female Balb/c mice	Increased rate of apoptosis when treated in the combined form of nisin-doxorubicin	Preet et al. (2015)
2016	*Streptoverticillium cinnamoneus*	Duramycin	Multicancer approach	Pancreatic cell line AsPC-1	Induces release of Ca^2+^ from cancer cells, Induces necrosis in cancer cells	(Broughton et al., 2016)
2017	*Lactococcus lactis*	Nisin	Colorectal cancer	SW480	Upregulation of Bax/Bcl2 genes, cytotoxic effect against SW480 cells	Ahmadi et al. (2017)
	*Enterococcus faecium* por1	Enterocin-A	Colon cancer, gastric cancer, cervical cancer	HT29, Caco2, AGS, HeLa	Cell cycle arrest in sub-G and G1 phase, induction of apoptosis	Ankaiah et al. (2017)
	*Brevibacillus* sp. strain SKDU10	Lactosporulin10	Multicancer approach	HeLa, RWPE-1, HEK293T, HT1080, H1299	Dose-dependent cytotoxic activity by membrane disintegration against cancer cell lines	Baindara et al. (2017)
2018	*Enterococcus faecalis*	Enterocin Oe-342	Colon cancer	HCT-116	Cell cycle arrest in G2/M phase, and membrane blebbing along with shrinkage of cancer cells	(Al-Fakharany et al., 2018)
	*Enterococcus faecium* por1	Enterocin-B, Enterocin-A + B	Colon cancer, cervical cancer, gastric cancer	HT29, HeLa, and AGS	Induction of apoptosis by nuclear fragmentation	Ankaiah et al. (2018)
	*Lactococcus lactis*	Nisin Z	Skin cancer	Human malignant melanoma (A375)	The generation of reactive oxygen species, affects the energy metabolism and induction of apoptosis	Lewies et al. (2018)
	*Lactococcus lactis*	Nisin	Colorectal cancer	LS180, SW48, HT29 and Caco2	Downregulation of CEA, CEAM6, MMP2F and MMP9F genes in all cell lines. Suppression of CEA protein expression	Norouzi et al. (2018)
	*Lactococcus lactis*	Nisin	Neuroblastoma	IMR-32, Neuroblastoma membrane model	Inhibition of IMR-32 cell proliferation by increasing the cell membrane fluidity	Prince et al. (2019)
2018	*Lactococcus lactis*	Nisin	Astrocytoma	SW1088 cell line	Inhibition of cell proliferation in a dose-dependent fraction	Zainodini et al. (2018)
2019	*Streptomyces azureus* ATCC 149215	Thiostrepton	Breast cancer	MCF-7	Suppression of FOXM1protein	Kongsema et al. (2019)
2020	*Enterococcus thailandicus*	Enterocin LNS18	Liver cancer	HepG2	Cell cycle arrest in G0 phase, increased production of ROS and downregulation of HepG2 markers	Al-Madboly et al. (2020)
2021	*Streptoverticillium cinnamoneus*	Duramycin	Liver cancer	MCA-RH 7777	Increased production of ROS, and induction of apoptosis	Yang et al. (2021)
	*Enterococcus faecium*	Enterocin 12a	Osteosarcoma, lung cancer, colon cancer, cervical cancer	MG-63, A549, HCT-15 and HeLa	Dose-dependent inhibition of cancer cell lines and induction of apoptosis through morphological alterations	Sharma et al. (2021)
2022	*Lactococcus lactis*	Nisin	Liver cancer	HuH-7 and SNU182	Downregulated genes responsible for epithelial-to-mesenchymal transition	Balcik-Ercin and Sever (2022)
2023	*Lactobacillus plantarum*	Plantaricin BM-1	Colorectal cancer	SW480	Induction of apoptosis through caspase-dependent pathway, downregulation of genes involved in TNF, NF-κB, and MAPK signalling pathway	(Wang et al., 2023)

### 3.3 Exopolysaccharides

Exopolysaccharides are biopolymers that microorganisms synthesize during their growth and metabolism (Welman and Maddox, 2003). Production of EPS can be varied from microorganism based on the monosaccharide composition and with the degree of branching. EPS is classified into homo-polysaccharide (containing the same monosaccharide units like dextran and cellulose) and hetero-polysaccharide with varying monosaccharides (xanthan) (Zhou et al., 2019). Synthesis of EPS is distinctly a strain-specific behavior and relies on several factors like the media composition, pH, and temperature (Behare et al., 2013). EPS is widely used in food industries as a stabilizing, emulsifying, and water-binding agent (Singh and Saini, 2017). The EPS of probiotic bacteria is found to exhibit anti-oxidative, anti-aging, anti-biofilm, and immunomodulatory effects as well as anti-tumor activity at *in vitro* and *in vivo* conditions (Di et al., 2017; Wang et al., 2019).

EPS has gained importance in scientific research due to its diverse properties like adherence towards intestinal epithelium and inhibition of pathogenic microbes in the gastrointestinal environment (Jurášková et al., 2022). Apart from anti-microbial, anti-biofilm, and anti-inflammatory, EPS derived from probiotic strains have been evaluated for their anti-cancer activity in various *in vitro* and *in vivo* conditions as shown in Table 4.

**TABLE 4:** Effect of EPS of probiotics against cancer.

Year	Probiotic strain	Cancer type	Study subject (cell lines/test animals)	Outcome	References
2010	*Lctobacillus acidophilus* 606	Colon cancer	HT-29	Downregulation of genes like Beclin-1, GRP78 and Bcl-2	Kim et al. (2010)
2011	*Lactobacillus casei* 01	Colon cancer	HT-29	Dose-dependent cytotoxicity against HT-29 cells	Liu et al. (2011)
2013	*Bacillus amyloliquefaciens*	Gastric cancer	MC-4, SGC-7901	Dose-dependent cytotoxicity against cancer cells with morphological disruptions including cell shrinkage and nucleus fragmentation	Chen et al. (2013)
	*Lactobacillus plantarum* NRRL B-4496	Multicancer approach	MCF-7, HepG2, Caco, HCT116, Hep G2	Inhibition of proliferation of cancer cells in a dose-dependent manner	Haroun et al. (2013)
2014	*Lactobacillus helvictus* MB2-1	Gastric cancer	BGC-823	Time-dependent inhibition of cell proliferation	Li et al. (2014)
	*Lactobacillus plantarum* 70,810	Gastric, Liver and colon cancer	Hep G2, BGC-823, HT- 29	Concentration-dependent inhibition of tumor cell growth	Wang et al. (2014)
	*Bacillus thuringiensis* S13	Lung cancer	A549	Cytotoxic activity against lung cancer cell line A549	(Karuppiah et al., 2014)
2015	*Lctobacillus acidophilus* MTCC 10307	Colorectal cancer	HCT-15, CaCo2	Suppression of VEGF, HIF-1α and upregulation of HIF-2α, PAI-1, TIMP-3 and HO-1	Deepak et al. (2016)
2017	*Bacillus flexus*	Liver cancer	Hep G2	Cytotoxic activity against cancer cell line Hep G2	Abdelnasser et al. (2017)
	*Bacillus amyloliquefaciens* 3MS 2017	Breast cancer, Prostate cancer	MCF-7, PC-3	Concentration-dependent inhibition of MCF-7 and PC-3 cell growth, inhibition activity against cyclooxygenases enzyme (COX-2)	El-Newary et al. (2017)
	*Lactobacillus gasseri*	Cervical cancer	HeLa	Upregulation of BAX and Casp3 gene, increase in IL-10 production, and decrease in TNF- α production	Sungur et al. (2017)
	*Lactobacillus casei* SB27	Colon cancer	HT-29	Upregulation of BAX, BAD, Casp 3, and Casp 8 genes. Induction of apoptosis by morphological disruption	Di et al. (2017)
	*Lactobacillus plantarum* NCU116	Colon cancer	CT-29 (Mouse cell line)	Upregulation of pro-apoptotic genes (Fas, Fasl, and c-Jun) and suppression of CT26 cell proliferation by Fas/Fasl-mediated apoptotic pathway	Zhou et al. (2017)
2018	*Lactobacillus acidophilus* 20,079	Breast cancer, Colon cancer	MCF-7, CaCo-2	Restricted the proliferation of cancer cells, upregulation of genes like p 53 and IKaB	El-Deeb et al. (2018)
	*Bacillus velezensis* MHM3	Breast cancer	MCF-7	Induction of apoptosis by activation of caspase-3, downregulation of Bcl2 gene, and increase the production of cyt c	Mahgoub et al. (2018)
	*Streptococcus thermophilus* CH9	Liver cancer	Hep G2	Induction of apoptosis with morphological alterations	Sun et al. (2018)
2019	*Lactobacillus casei, Lactobacillus paracasei*	Colon cancer	HT-29	Time-dependent induction of apoptosis by DNA fragmentation	Mojibi et al. (2019)
	*Lactobacillus kefri* MSR101	Colon cancer	HT-29	Induction of apoptosis by upregulation of Cyt-c, BAX, BAD, caspase3, caspase8 and caspase9, downregulation of Bcl-2	Riaz Rajoka et al. (2019)
	*Lactobacillus delbrueckii* ssp. *bulgaricus*	Colon cancer	HT-29	Time independent inhibition of cell proliferation by apoptosis, upregulation of BAX, caspase3, caspase9 and downregulation of Bcl2, survivin	Tukenmez et al. (2019)
	*Bifidobacterium breve* lw01	HNSCC	SCC15, CAL 27, WSU-HN6	Inhibition of cell proliferation in dosage-dependent manner, cell cycle arrest and promotion of apoptosis	(Wang et al., 2019)
	*Lactobacillus fermentum* YL-11	Colon cancer	HT-29, CaCo-2	Inhibition of cell growth and proliferation, cytotoxic activity against cancer cells	Wei et al. (2019)
2020	*Bacillus amyloliquefaciens* 3M 2017	Breast cancer	Sprague-Dawley rats	Inhibition of COX-2 gene expression, inhibition of growth-limiting enzymes like aromatase and ATPase	Ibrahim et al. (2020)
2021	*Lactiplantibacillus plantarum* 12	Colon cancer	C57BL/6 mice, HT-29	Induction of apoptosis by activation of caspase cascade, upregulation of caspase -8, caspase-9, and caspase-3, and downregulation of PCNA	(Ma et al., 2021)
	*Bacillus* sp NRC5	Breast cancer, Prostate cancer	MCF-7, PC3, and Albino female mice	Inhibition of COX-2 gene, reduction of tumor weight in mice	Mohamed et al. (2021)
2022	*Lactobacillus delbrueckii* ssp. *Bulgaris* DSMZ 20081	Multi cancer approach	HEK 93, CaCo2, HepG2, MCF-7	Cytotoxic effect against cancer cells, upregulation of BAX, Caspase 3, Caspase 8, p53, and downregulation of BCl-2, MCL1, and vimentin genes	Khalil et al. (2022)
	*Lactobacillus pantheris* TCP102	Colon cancer, gastric cancer, and ovarian cancer	HCT-116, A-2780, BCG-803	Suppression of cell proliferation in cell lines, production of nitric oxide	Sheng et al. (2022)
2023	*Bacillus subtilis*	Breast cancer cells	MCF-7, T47D, MDA-MB-231, MDA-MB-453, MDA-MB-468 ZR-75-30, HCC1428, and BT549	Upregulation of pro-inflammatory pathways like STAT1 and NF-kB.IKKβ, induction of apoptosis, and cell cycle arrest G1/G0 phase	Nguyen et al. (2023)
	*Lactiplantibacillus plantarum* YT013	Gastric cancer	AGS	Concentration-dependent induction of apoptosis, upregulation of BAX, BAD, Caspase-3, Caspase-8, and Caspase-9, and downregulation of Bcl2	Zhang et al. (2023)

### 3.4 Short-chain fatty acids (SCFAs)

SCFAs belong to the metabolite produced by the probiotic bacteria as a result of metabolism and they are aliphatic compounds with 1-6 carbons. The gut microbiome has the potential to generate large amounts of SCFA from the available fermented carbohydrates and non-digestible components present in the gastrointestinal environment (Mirzaei et al., 2021). SCFAs are absorbed by the process of simple diffusion and active transport by transporters present over the membranes of all tissues and cells including the immune cells (Kim et al., 2014). SCFAs that are not taken up by the colonocytes are transported over the basolateral membrane enter the blood circulation and affect other cells directly (den Besten et al., 2013). Lack of SCFA production may lead to the pathogenicity of several diseases like asthma, neurological disease, and cancer (Tan et al., 2014). The most abundant SCFAs are acetate, propionate and butyrate, produced by *Clostridium, Propionibacterium*, and *Lactobacillus* species. In recent days, SCFAs have been developed and employed as immunomodulatory therapeutics as it has several advantages compared to the microbe-based methods (Feitelson et al., 2023). SCFAs interplay between the gut and different organs through systemic circulation (Tsvetikova and Koshel, 2020). Mainly SCFA-related effects are associated with two pathways: activation of GPCR (G-protein coupled receptors) and suppression of histone deacetylases (Carretta et al., 2021). SCFA, especially butyrate has been widely studied against cancer as it is believed to be involved in anti-cancer activity by altering cellular responses to the metabolism and oxidative stress (Vrzáčková et al., 2021). Several studies report that SCFA induces apoptosis in cancer cells by disrupting membrane potential, enhancing the expression of GPCR molecules, and mitochondrial depolarization (Table 5).

**TABLE 5:** Effects of SCFAs against cancer.

Year	Probiotic strain	SCFA	Cancer type	Study Subject (cell lines/test animals)	Outcome	References
2002	*Propionibacterium acidipropionici, Propionibacterium freudenreichii*	Propionate, acetate	Colorectal cancer	HT-29	Induction of apoptosis by with loss of mitochondrial transmembrane potential, and nuclear chromatin condensation	Jan et al. (2002)
2005	*Butyrivibrio fibrisolvens*	Butyrate	Colorectal cancer	Male Jcl: ICR mice (4 weeks old)	Increased number of NK and NKT cells, decreased β-glucuronidase activity	Ohkawara et al. (2005)
2006	*Propionibacterium freudenreichii*	Propionate, acetate	Colorectal cancer	HT-29	Induction of cell cycle arrest in the G2/M phase, mitochondrial depolarisation, ROS accumulation and destruction in ATP levels	Lan et al. (2007)
2013	*Pediococcus pentosaceus* FP3, *Lactobacillus salivarius* FP25, and *Lactobacillus salivarius* FP35	Butyric and propionic acid	Colon cancer	CaCo-2	Dose-dependent induction of cancer cell death, induction of apoptosis by caspase-3 activity	Thirabunyanon and Hongwittayakorn (2013)
	*Clostridium butyricum*	Butyrate	Colon cancer	C57BL/6 mice	Upregulation of Foxp 3 gene in colonic T_reg_ cells	Furusawa et al. (2013)
2015	*Lactobacillus fermentum* NCIMB 5221, *Lactobacillus fermentum* NCIMB 2797	Acetate, butyrate, and propionate	Colon cancer	CaCo-2	Time-dependent inhibition of CaCo-2 cell proliferation	Kahouli et al. (2015)
2018	*Propionibacterium freudenreichii*	Acetate, propionate	Colon cancer	HT-29	Cytotoxic effects against CRC cells, cell cycle arrest at G2/M phase	Casanova et al. (2018)
2020	*Butyricicoccus pullicaecorum*	Butyrate	Colorectal cancer	SW480, SW620 BALB/cByJNarl male mice (4–6 weeks)	Upregulation of SLC5A8 expression in cell line as well as mice, decrease in tumor progression in mice	Chang et al. (2020)
	*Clostridium butyricum*	Butyrate	Colorectal cancer	HCT 116, CaCo-2, HCT-8, *Apc* ^min/+^ mice (4-weeks-old)	Suppression of tumor development by altering the Wnt/β-catenin signaling pathway, increase in expression of GPR43 and GPR109A	Chen et al. (2020)
2021	*Escherichia coli*	Butyrate	Colorectal cancer	HT-29, BALB/cAnN.Cg male mice (4-weeks old)	Induction of apoptosis by cell cycle arrest at G1 phase, induction of mitochondrial apoptotic pathway Reduction of 70% tumor volume in mice	Chiang and Hong (2021)
	*Escherichia coli* KUB-36	Acetic acid, butyric acid	Colon cancer, breast cancer	HT-29, MCF-7	Dose-dependent cytotoxic effect against cell lines, increased expression of IL-10 gene	Nakkarach et al. (2021)
	*Butyricicoccus pullicaecorum*	Butyrate	Urinary bladder cancer	HT 1376	Increase in expression of GPR43, FABP4 and BLCAP genes	Wang et al. (2021)
2022	*Lactoplantibacillus plantarum* S2T10D	Butyrate	Colon cancer	HT-29	Downregulation of cyclin D1 gene expression, cell cycle arrest at G2/M phase	Botta et al. (2022)
2022	*Lactobacillus paracasei* SD1, *Lactobacillus rhamnosus* SD11	Butyrate	Colon cancer	CaCo-2, HIEC	Dose as well as time-dependent inhibition of cancer cell growth, accumulation of butyrate in the nucleus leading to apoptosis	Thananimit et al. (2022)

### 3.5 Conjugated linoleic acids (CLA)

Probiotics are capable of hydrogenation of long-chain fatty acids. In the process of hydrogenation, the free fatty acid is converted into its conjugate form (Dubey et al., 2012). Probiotic strains belonging to *Bifidobacteria* and *Lactobacillus* species are the predominant CLA producers that are widely used in several functional foods (Ghosh and George, 2023). Apart from these main groups of probiotics, species of *Propionibacterium, Streptococcus*, and *Enterococcus* present in the intestinal gut flora also produce fewer amounts of CLA (Palla et al., 2021). CLA production was also identified in the *Pediococcus* strain apart from well-known probiotics strains (Dubey et al., 2012). CLA has been shown to possess numerous health benefits like anti-diabetic, anti-inflammatory, anti-atherogenic, and anti-carcinogenic properties in both *in vitro* and *in vivo* studies (Ewaschuk et al., 2006). Scientific evidence depicts that CLA can inhibit the proliferation and growth of cancer cells and induce apoptosis (Table 6).

**TABLE 6:** Role of CLAs against cancer.

Year	Probiotic strain	Cancer type	Study subject (cell lines/test animals)	Outcome	References
2006	*Bifidobacterium breve*	Colon cancer	HT-29, CaCo-2	Suppression of cancer cell proliferation	Coakley et al. (2006)
	VSL-3	Colon cancer	HT-29, CaCo-2	Induction of apoptosis, upregulation of PPARγ expression	Ewaschuk et al. (2006)
2007	*Propionibacterium acnes*	Colon cancer	SW480	Concentration-dependent growth inhibition of cancer cells, suppression of cell proliferation	Rosberg-Cody et al. (2007)
2009	*Bifidobacterium breve* NCIMB 702258	Colon cancer	SW480	Suppression of growth of SW480 cells	Coakley et al. (2009)
2016	*Pediococcus pentasaceus* GS4	Colon cancer	HCT-116	Downregulation of NF-κB and p-Akt, induction of apoptosis, and inhibition of cell proliferation	Dubey et al. (2016)
	*Bifidobacterium breve* DPC6330	Colon cancer	SW480	Downregulation of Bcl-2 gene expression, suppression of cancer cell proliferation	Hennessy et al. (2016)
	*Lactobacillus plantarum*	Breast cancer	MDB-MB-231	Suppression of NF-κB pathway, degradation of proteasome of IқBα, upregulation of Bax gene, and release of Cyt-C from mitochondria	Kadirareddy et al. (2016)
2023	*Pediococcus pentasaceus* GS4	Colon cancer	HCT-116	Reduced expression of COX-2 and 5-LOX, mitochondrial membrane depolarization, and increase in caspase 1p10 expression	Dubey et al. (2023)

### 3.6 Peptidoglycan and other metabolites

Excepting the major postbiotic components, some structural compounds present in the probiotic bacteria also play a vital role in contributing to host health. Peptidoglycan, commonly known as murein is one of the major bacterial cell wall components which maintain the morphology of cells (Dramsi et al., 2008). Some researchers reported that these molecules possess anticancer activity by altering apoptotic gene expressions and inhibiting cell growth (Table 6). However, PG as a postbiotic component may augment inflammation. PG, being a part of PAMP (pathogen-associated molecular pattern)/or DAMP (damage-associated molecular pattern) may induce components of host-PRR (pattern recognition receptors), mainly via TLR-2, TLR-4 (toll-like recptors) to induce inflammation by innate immune cells, macrophages, neutrophils, dendritic cells to sustain inflammation at the local tissue microenvironments which may cause host-tissue damages. It may induce, and activate immune cells to release of proinflammatory cytokines like IL-1β, IL- 6, IL-8, and TNF α. Thus, it bears immunological limitations in its use for benefitting host health. Considering the wide range of applications of extracellular vesicles (EV), EVs from probiotic bacteria were employed against cancer cell lines. EVs are membrane-bound components that are spherical, consist of a lipid bilayer, and transfer genetic materials through the process of horizontal gene transfer (Ghosh, 2024; Ahmadi Badi et al., 2017). EVs contain proteins, DNA, RNA, glycolipids, polysaccharides, enzymes, and some endotoxins (Chelakkot et al., 2018). EVs were found to block the cell cycle and suppress cell proliferation (Table 7).

**TABLE 7:** Effects of heat-killed cells, peptidoglycan, and other cellular components of probiotics against cancer.

Year	Probiotic strain	Component	Cancer type	Study subject (cell lines/test animals)	Outcome	References
2002	*Bifidobacterium longum, Lactococcus lactis* ssp.*lactis*	Peptidoglycan	Colon cancer, gastric cancer	DLD1, SNU-1	Inhibition of cancer cell line proliferation	(Kim et al., 2002)
2008	*Lactobacillus casei*	Peptidoglycan	Colitis-associated cancer (Colon cancer)	Female BALB/c mice (8weeks old)	Downregulation of IL-6 gene expression	Matsumoto et al. (2009)
2015	*Lactobacillus paracasei subp. Paracasei* X12	Peptidoglycan	Colon cancer	HT-29	Regulates Ca^2+^ release from the endoplasmic reticulum into the cytoplasm, induction of apoptosis, upregulation of HMGB1 protein, and translocation of calreticulin which influences malignant transformation	Tian et al. (2015)
2016	*Bacillus lentus*	Membrane vesicles	Colon cancer	HCT-116	Activation of caspase-9, caspase-3, upregulation of BAX gene, downregulation of Bcl-2 gene and promotes the release of Cyt c from mitochondria	Yang et al. (2016)
2017	*Lactobacillus plantarum*	Peptidoglycan	Skin cancer	C57BL/6 female mice	Decreases VEGF levels and cytotoxic towards cancer cells	Aintablian et al. (2017)
	*Lactobacillus acidophilus* ATCC 4356	Peptidoglycan	Colon cancer	HT-29	Dose-dependent inhibition of HT-29 cell growth, induction of apoptosis	He et al. (2017)
2018	*Lactobacillus paracasei subp. Paracasei* M5	Peptidoglycan	Colon cancer	HT-29	Cytotoxicity against cancer cells, downregulation of Bcl-xl gene, decreased Cyt c level in cytosol, induction of apoptosis by caspase-3 dependent pathway	Wang et al. (2018)
2019	*Lactobacillus paracasei* IBRC_M10784, *Lactobacillus brevis* IBRC_M1079	Heat killed cells	Colon cancer	HT-29	Induction of apoptosis, upregulation of BAX gene, caspase-3, caspase-9and downregulation of Bcl-2, release of Cyt c leading to activation of mitochondrial pathway	Karimi Ardestani et al. (2019)
2022	*Lactobacillus brevis* KU15176	Heat killed cells	Gastric cancer	AGS	Increased the expression of BAX, caspase-3, and caspase-9, DNA breakage, and induction of apoptosis	Hwang et al. (2022)
2022	*Lactobacillus casei* MG4584, *Lactobacillus reuteri* MG5346	Heat killed cells	Colon cancer	RKO BALB/c mice	Increased the expression of caspase-3, caspase-9, and caspase-7, activation of PARP, and activation of intrinsic apoptotic pathway	(Kim et al., 2022)
2023	*Limosilactobacillus* *Fermentum* LAC92	Peptidoglycan	Colon cancer	HCT-116	Anti-proliferative effects and induction of apoptosis	Fuochi et al. (2023)
2024	*Lentilactobacillus buchneri*	Extracellular vesicles	Colon cancer, gastric cancer	HT-29, AGS	Cell cycle arrest at G0/G1 Phase, upregulation of BAX, caspase-3 and caspase-9 gene	Abedi et al. (2024)
	*Lacticaseibacillus paracasei* PC-H1	Extracellular vesicles	Colon cancer	HCT-116 BALB/c mice	Downregulation of HIF-1α, GLUT1, and LDHA gene expression, suppression of cell proliferation	Shi et al. (2024)
	*Lactobacillus plantarum subsp. plantarum* NBRC 15891	Heat killed cells	Colon cancer	HT-29	Suppression of IL-8 production in cell line	Yamasaki-Yashiki et al. (2024)

Apart from above mentioned bioactive postbiotics components, heat-killed (HK) probiotic cells have created experimental data in the field of medicine. HK cells exist in inactive form achieved by incomplete autoclaving and by cell freezing technique (Taverniti and Guglielmetti, 2011). These heat-killed cells showcased competency for adhesion sites against pathogens in a Caco-2 cell line model (Singh and Saini, 2017). Additionally, several findings validate that heat-killed cells have the potential to modulate host health and as a competing anti-cancer agent (Table 7)

## 4 Mechanism of action of postbiotics against cancer

### 4.1 CFS - Mechanism of action

CFS is a result of simple preparation by cultivating live probiotics in media, centrifugation, and filter sterilization which exhibits multiple probiotic characteristics. Study with CFSs of probiotics (*Bifidobacterium adolescentis* SPM0212, *Lactobacillus rhamnosus* GG, *Lactobacillus delbrueckii*, *Bacillus polyfermenticus* KU3, *Lactobacillus fermentum* NCIMB5221, *Lactobacillus reuteri* NCIMB 701359; *Lactobacillus pentosus* B281, *Lactoplantibacillus plantarum* B282; *Lactobacillus casei* (SR1,SR2)*, Lactobacillus paracasei* (SR4); *Lactobacillus salivarius* and many other strains) using different cancer cell lines (e.g., Caco-2, HCT-116, HT-29, HeLa, LoVo, SW480, SW620, AGS, and MCF-7, CRL-1831and other cell lines related to respective cancer) demonstrated pathophysiological, cell biological and immunological impact to abrogate the cancer progression, metastasis by induction of apoptosis and inhibition of cancer cell proliferation; downregulation of cyclin D1 (cell cycle arrest in G1-phase), cyclooxygenase-2, protein kinase B and NF- κB activation; downregulation of Bcl-2 gene, cleavage of caspase-9, caspase-3, and PARP; downregulation of MMP2, MMP9, and suppression of the VEGF pathway; and upregulation of BAX, BAD genes (Table 2). CFAs of probiotic strains demonstrate a complete anti-cancer interaction with studied cell lines. Similarly, results obtained from animal studies using the C57BL/6 mouse (Liu et al., 2021), and MCF-7 xenograft mouse (Behzadi et al., 2021) model reveal that probiotic CFSs have the demonstrable potential for inhibition of cell proliferation and reduction in weight of tumor; and inhibition of epithelial-mesenchymal transition (EMT) by regulating Wnt/β-catenin pathway.

### 4.2 Bacteriocins - Mechanism of action

**FIGURE 3:**
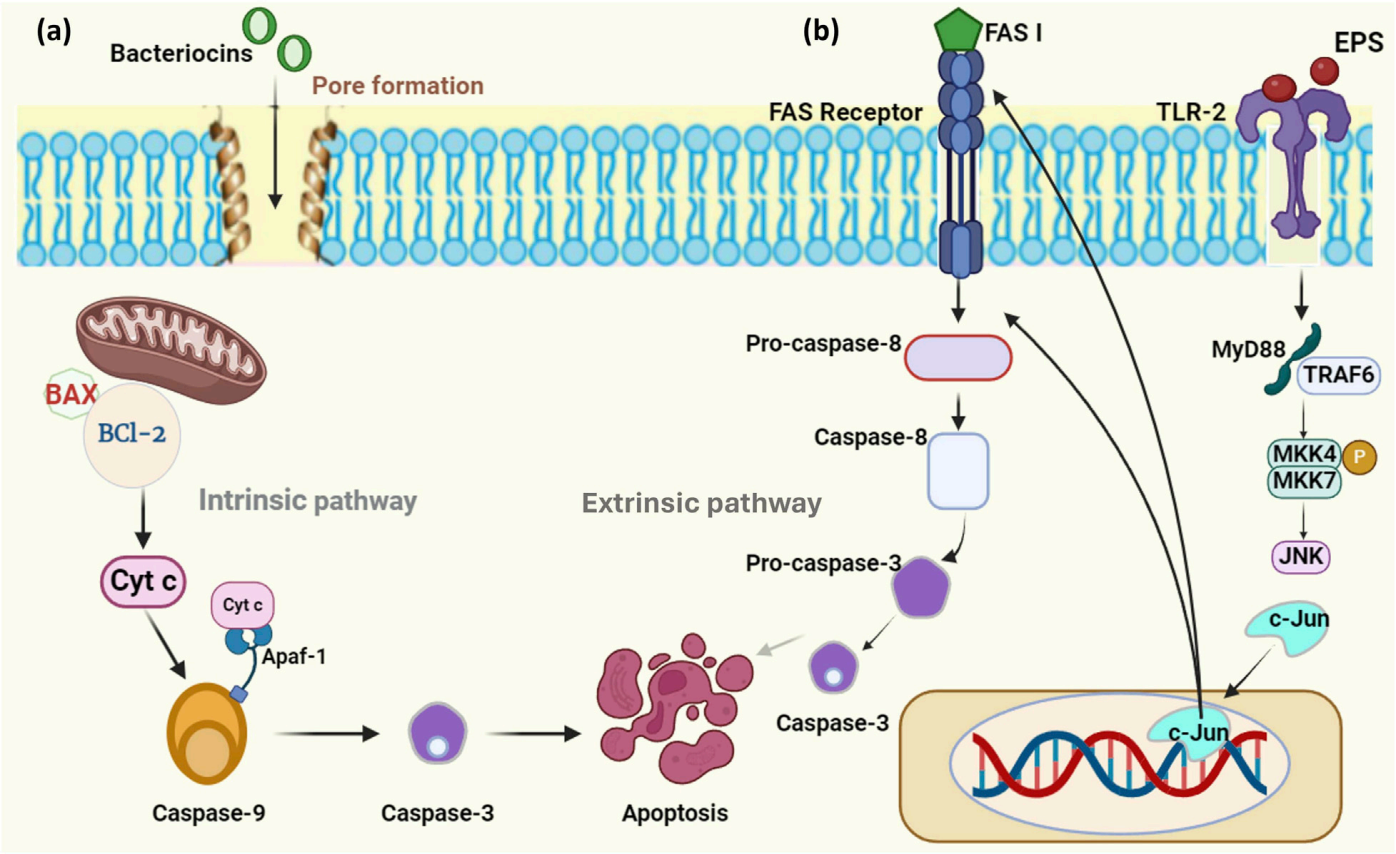
Mechanism of action of bacteriocin and EPS on cancer. **(A)** Bacteriocins downregulate the expression of the BCl-2 gene, upregulate the BAX gene, and induce mitochondria to release Cyt c which activates the intrinsic pathway resulting in apoptosis. **(B)** EPS binds to the TLR and activates c-Jun which helps in the activation of the caspase-8-mediated extrinsic apoptotic pathway. (Figures were generated using BioRender.com).

Bacteriocins were identified to induce apoptosis in cancer cells through cancer signaling pathways. As bacteriocins possess a cationic, amphiphilic, and hydrophobic nature, they target tumor cells resulting in apoptosis (Wang et al., 2024). Nisin, the class I lantibiotics, induced apoptosis in cancer cells by regulating the intrinsic pathway, intervened by mitochondria. Also, BCL-2 (B-cell lymphoma 2) family proteins such as Bcl-2 and BAX gene expression were altered in colon cancer cell lines (Ahmadi Badi et al., 2017). Normally, Bcl-2 protein expression is observed to be higher in cancer cells compared to that of normal cells. Apart from that, Bcl-2 family proteins act as an obstacle to apoptosis, develop resistance to the therapy, and in tumor development (Campbell and Tait, 2018). Generally, cancer cells are found to be resistant to apoptosis, on that note a study reported that, mechanisms that induce apoptosis begin with the release of cytochrome c (Cyt c) from the mitochondria and persuade ER to produce calcium. Both of these molecules play a vital role in apoptosome formation, activating cell surface death receptors and initiating caspase-dependent pathways (Joo et al., 2012). Cyt c mainly functions as an electron carrier during the mitochondrial respiratory chain, interacts with Apaf-1 (apoptotic protease activating factor-1) that exists in the cytosol, and enables it to form apoptosomes leading to activation of caspase-9 and caspase-3 that implements programmed cell death (Figure 3A) (Elena-Real et al., 2018). In another study, nisin treatment in human colon cancer cell lines showcased the altered expression of CEA (Carcinoembryonic antigen) and matrix metalloproteinase (MMP) genes (Norouzi et al., 2018). These MMPs are found to be potential modulators in the development of cancer, which can directly involve cancer signaling pathways and control apoptosis (Kessenbrock et al., 2015).

Some of the studies pointed out that enterocin resulted in cell cycle arrest on human cancer cell lines (Anakiah et al., 2017; Al-Fakharany et al., 2018; Al-Madboly et al., 2020). Cell cycle arrest is considered the emerging approach against cancer, as this mechanism supports tumor cells to restore their damaged DNA. Thus, negating cell cycle checkpoints before the DNA repair mechanism can lead to the activation of apoptotic cascade pathways that result in cell death would be a promising approach in cancer therapy (Schwartz et al., 2005).

### 4.3 Exopolysaccharides - Mechanism of action

Exopolysaccharides comprise proteins, extracellular DNA, lipids, and a major number of polysaccharides which enables them a wide range of health-benefiting properties (Di Martino, 2018). Several studies report that probiotic exopolysaccharides inhibit cancer cells without affecting normal cells, compared to synthetic drugs. There are various possible mechanisms of EPS to act upon cancer cells like induction of apoptosis, prevention of cell proliferation, and improvement of the host immune system (Angelin and Kavitha, 2020). Apoptosis is associated with two major caspase-dependent pathways known as intrinsic and extrinsic pathways. The differences in these two depend on the genes and proteins that are involved in driving the pathway. In intrinsic pathways, caspase-3, caspase-9, BAX, and, BCl-2 genes are expressed, whereas the extrinsic pathway involves caspase-8 and caspase-10 expression (Jan and Chaudhry, 2019). Activation of caspase-3 is a sign indicating that the cancer cells have undergone cell shrinkage, chromatin condensation, and nuclear fragmentation effectively (Jung et al., 2001). Previously it was found that EPS from *L. gasseri* was able to inhibit the proliferation of HeLa cells by upregulation of BAX and caspase three gene expression which leads to activation of apoptosis (Sungur et al., 2017). EPS derived from *L. plantarum* NCU116 witnessed an increase in the expression of pro-apoptotic genes like Fas, FasL, and c-Jun along with TLR-2 in mouse intestinal cells (Figure 3B) (Zhou et al., 2017). These Fas genes known as the first apoptosis signal along with its receptor mainly trigger the extrinsic pathway of apoptosis that is responsible for the suppression of tumors, so upregulation of the genes can induce apoptosis (Peter et al., 2015). EPS of *L. delbrueckii* ssp. *Bulgaris* exhibited upregulation of the p53 gene along with other caspase genes that are involved in inducing apoptosis (Khalil et al., 2022). p53 acts as a tumor suppressor gene, involved in inducing cell cycle arrest and a nuclear transcription factor possessing pro-apoptotic function. This gene is also found in high levels in patients suffering from cancer with mutant types of p53 (Ozaki and Nakagawara, 2011). Hence due to their disparate mechanisms listed in Table 3 in treating cancer cells, these can be employed in the treatment of cancer with evidence of phase trials.

### 4.4 Conjugated linoleic acid – mechanism of action

**FIGURE 4:**
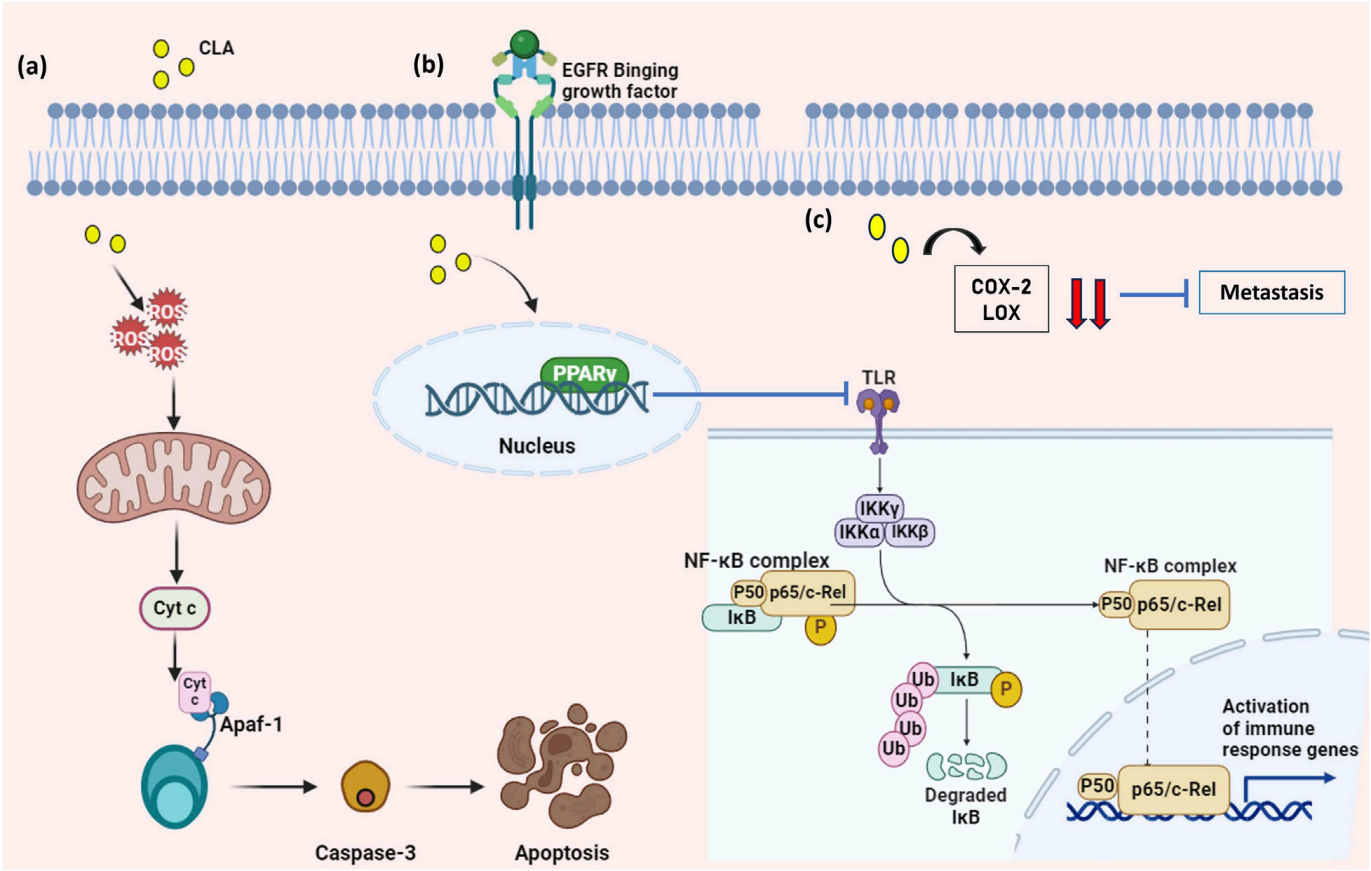
Mechanism of action of CLA against cancer. **(A)** CLAs produce ROS that stimulates the production of Cyt c which activates the caspase-cascade pathway leading to apoptosis. **(B)** CLAs upregulate the expression of PPAR γ in the nucleus which inhibits the NF- κB signaling pathway and **(C)** CLA downregulate the expression of COX and LOX genes that are involved in metastasis. (Figures were generated using BioRender.com).

Even though CLA has been well known for its wide range of applications, there are fewer studies involving CLA derived from probiotics against cancer. A study found that CLA extracted from *L. plantarum* exhibited anti-cancer activity in mammalian breast cancer cell lines by suppressing the NF- κB pathway and then by upregulation of the BAX gene leading to an apoptotic pathway (Kadirareddy et al., 2016). Apart from this, CLA produced by *P. pentosaceus* GS4 possesses anti-cancer activity in colon cancer cell line (HCT-116) by downregulation of NF- κB and inducing apoptosis (Dubey et al., 2016). This NF- κB pathway is mainly involved in the development and progression of tumors, cellular immunity, inflammation, and regulation of cell differentiation. NF- κB promotes the expression of genes of the Bcl-2 family, caspase-8 inhibitor proteins, and other apoptosis-inhibiting proteins which primarily function by preventing apoptosis of a cell (Figure 4A) (Xia et al., 2018). Moreover, the NF- κB signaling pathway was identified as contributing to metastasis and also preventing the process of epithelial-to-mesenchymal transition (EMT) (Hoesel and Schmid, 2013).

Apart from these pathways, genes like COX-2 and LOX are identified and reported that they induce metastasis as well as cancer-supporting mechanisms. COX-2 gene is produced by fibroblasts that are associated with cancer and also by type 2 macrophage cells, which mainly promotes proliferation, apoptotic resistance, invasion, and metastasis in cancer cells (Hashemi Goradel et al., 2019). Along with the COX-2 gene, the LOX gene plays a vital role in inflating cancer cell proliferation, angiogenesis, and metastasis. CLA from *P. pentosaceus* GS4 has been reported to downregulate both the COX-2 and 5-LOX gene expression in the colon cancer cell line (HCT-116) (Figure 4C). Also, the upregulation of PPAR γ, a nuclear receptor that acts as a regulator of cell metabolism and functions as an inhibitor of cancer cell growth. PPAR γ causes oxidative stress and the flow of electrons that promote apoptotic cascades and some effects on mitochondria-mediated cell metabolism (Figure 4B). (Ghosh and George, 2023). CLA that is produced by the *P. pentosaceus* GS4 possesses biohydrogenation ability which modulates cancer by modulation of PPAR γ concerning anti-proliferative ability (Dubey et al., 2023).

### 4.5 Short-chain fatty acids – mechanism of action

**FIGURE 5:**
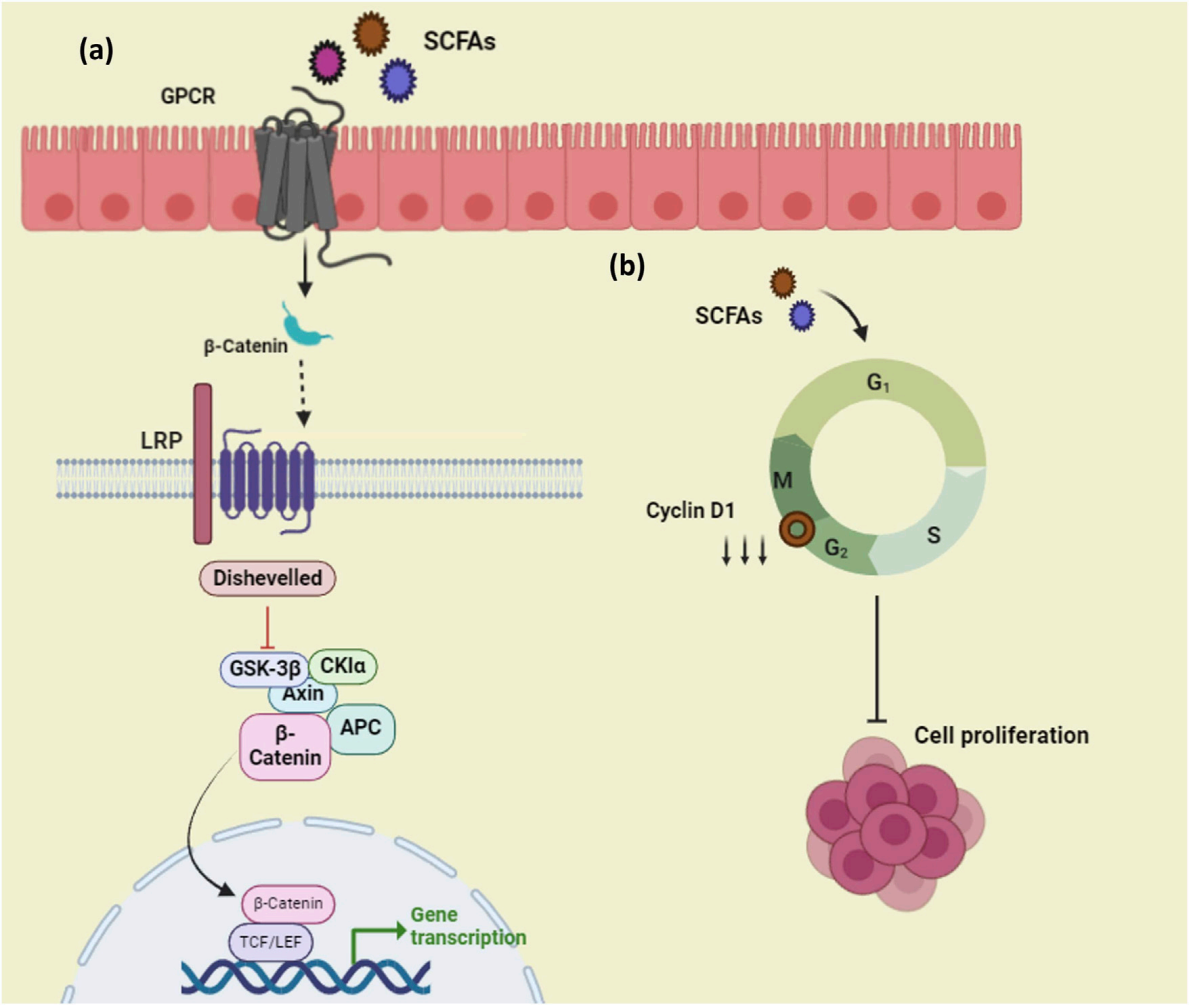
Mechanism of action of SCFAs on cancer, **(A)** SCFAs act as ligands to the GPCR present at the membrane layer and modulate the Wnt- β catenin pathway, **(B)**SCFAs downregulate the expression of CDK genes and inhibit cell proliferation of cancer cells. (Figures were generated using BioRender.com).

SCFAs that are produced from probiotics possess anti-proliferative, apoptotic, and cell cycle arrest properties over cancer cells as well as contribute to prohibiting carcinogenesis in the gut (Tripathy et al., 2021). Harnessing SCFAs against cancer can pave the way to getting rid of the crisis because of its impact on the expression of multiple genes and their related pathways that are relevant to cancer. It is found that GPR43 suppresses tumor growth by modifying the gut flora (Kong et al., 2022). SCFAs also function as ligands for G-protein coupled receptors (GPCR). There are receptors like GPR43 which specifically have a higher affinity for propionate and GPR109a for butyrate (Feitelson et al., 2023). SCFAs are found to upregulate the expression of β-catenin and regulate Wnt which may promote the differentiation of cancer cells as well as induce intestinal homeostasis (Jiang et al., 2019). Some studies suggest that these molecules can interfere with cancer pathogenesis before tumor formation by regulating Wnt and inhibiting proliferation (Feitelson et al., 2023). Butyrate from *Clostridium butyricum* was found to suppress the development of tumors by interfering with the Wnt/β catenin pathway and also by increasing the gene expression of GPR43 and GPR109A (Figure 5A). (Chen et al., 2020). Similarly, butyrate from *Butyricicoccus pullicaecorum* employed against urinary bladder cancer upregulated the expression of GPR43 resulting in the mitigation of cancer (Wang et al., 2021). Butyrate from *L. plantarum* S2T10D was found to suppress the expression of the cyclin D1 gene and also arrest the cell cycle at the G2/M phase (Figure 5B). (Botta et al., 2022). Cyclin D1 is one of the key regulators that performs a central role in the pathogenicity of cancer determining the cell proliferation and overexpressed in cases of cancer, whereas they are properly regulated in normal cells. Targeting this cyclin D1 could be a promising strategy to prevent tumor development (Montalto and De Amicis, 2020).

## 5 Future directions and conclusion

Cancer treatment in the modern era involves a dual approach based on the type of cancer and its associated characteristics like progression, area of localization, and metastasis (Ghosh and George 2023). Along with chemotherapy, other treatments are being developed with higher effectiveness and minimizing side effects to the host (Mármol et al., 2017). Probiotics and postbiotics have showcased their efficacy against various diseases and cancers with their role in vital processes like apoptosis induction, downregulation of tumor-inducing genes, suppression of cell proliferation, and prevention of metastasis (Sankarapandian et al., 2022). Growing shreds of evidence state that a combination of probiotics and postbiotics can be practiced as an adjuvant for patients undergoing chemotherapy (Lu et al., 2021). Significant research is in progress for employing probiotics and their bioactive metabolites (postbiotics) against cancer. Despite the wide usage of probiotics in treating different diseases, some side effects are caused in a small proportion of the population due to their uptake. In 2002, WHO-FAO released a report (http://www.fda.gov/ohrms/dockets/dockets/95s0316/95s-0316-rpt0282-tab-03-ref-19-joint-faowho-vol219.pdf) stating that “probiotics may be responsible for mainly four types of side effects” including systemic infections, deleterious metabolic activities, excessive immune stimulation in susceptible individuals, and gene transfer. Apart from these major effects, minor gastrointestinal symptoms like diarrhea, and other digestive problems are also reported after the uptake (Doron and Snydman, 2015). Probiotics have been reported to induce bacteremia, fungemia, localized infection, liver abscess, and endocarditis (Liu et al., 2024). Rather than live cells, these postbiotic components have created a cornerstone among researchers all over the globe due to their broad range of applications. In that case, bioactive compounds with anticancer properties, such as bacteriocins, EPS, SCFAs, and CLAs can be utilized as anticancer agents (Liu et al., 2021). Probiotics have been reported to induce bacteremia, fungemia, localized infection, liver abscess, and endocarditis (Liu et al., 2024). Rather than live cells, these postbiotic components have created a cornerstone among researchers all over the globe due to their broad range of applications. In that case, bioactive compounds with anticancer properties, such as bacteriocins, EPS, SCFAs, and CLAs can be utilized as anticancer agents (Liu et al., 2021). Despite their numerous properties, they possess some limitations like decreased bioavailability, and susceptibility against proteolytic enzymes in the GIT when they are orally administered. To overcome this lag, various strategies like encapsulation technology involving semi-synthetic techniques can improve their biological activity, stability, and also physiochemical activities (Xu et al., 2024). Even though there are numerous research articles, review articles, and products based on probiotics, there is countable proper evidence with clinical studies provided for the usage of probiotics with or without postbiotics for cancer prevention.

The present review highlights the involvement of postbiotic components from the potential probiotic strains employed against various types of cancer in both *in vitro* and *in vivo* studies along with their reported mechanism of action. From the above-reported studies, it is evident that different postbiotic components can be introduced in treating cancer as adjuvants that aid in decreasing the side effects caused by regular treatments. The rising trends of outcomes from the research are growing significantly with well-grounded data before recommending probiotics and postbiotics as alternative therapies for treating and preventing various forms of cancer. However, extensive research is needed to explore the anticancer efficacy of the specific or combined postbiotic-bioactive components as an alternative treatment strategy for preventing and controlling cancers.

## Abbreviations

BAX, Bcl-2 associated protein X; BAK, Bcl-2 homologous antagonist/killer; GLUT-1, Glucose transporter 1; LDHA, Lactate dehydrogenase A; Cyt c, Cytochrome c; VEGF, Vascular endothelial growth factor; IL, Interleukin; COX-2, Cyclooxygenase 2; 5-LOX, 5-lipoxygenase; NF- κB, Nuclear factor kappa B; PPAR, Peroxisome proliferator-activated receptors; GPR, G-Protein coupled receptor; ROS, Reactive oxygen species; Fas, First apoptosis signal; FOX M1, Forkhead Box Protein M1; CEA, Carcinoembryonic antigen; EMT, Epithelial-mesenchymal transition; TNF, Tumor necrosis factor; MMP, Matrix Metalloproteinases.
